# The impact of COVID-19 hospitalizations on nursing home admissions: a regional insight into long-term care and public health

**DOI:** 10.3389/fpubh.2025.1613684

**Published:** 2025-07-18

**Authors:** Alessandra Bandera, Marta Colaneri, Alessia Antonella Galbussera, Marta Canuti, Lucia Dall’Olio, Alessandro Nobili, Massimo Puoti, Giulia Carla Marchetti, Simone Piva, Pierluigi Plebani, Mario Raviglione, Andrea Gori, Danilo Cereda, Olivia Leoni, Ida Fortino, Maria Luisa Ojeda Fernandez, Pier Mannuccio Mannucci, Pasquale Agosti, Fabrizio Tediosi, Marta Baviera, Mauro Tettamanti

**Affiliations:** ^1^Infectious Diseases Unit, Fondazione IRCCS Ca’ Granda Ospedale Maggiore Policlinico, Milan, Italy; ^2^Department of Pathophysiology and Transplantation, University of Milan, Milan, Italy; ^3^Department of Biomedical and Clinical Sciences, Infectious and Tropical Diseases Operational Unit ASST Fatebenefratelli Sacco, “L. Sacco” University Hospital, Milan, Italy; ^4^Department of Health Policy, Istituto di Ricerche Farmacologiche Mario Negri IRCCS, Milan, Italy; ^5^Department of Veterinary and Animal Sciences, University of Copenhagen, Frederiksberg, Denmark; ^6^Centre for Multidisciplinary Research in Health Science (MACH), University of Milan, Milan, Italy; ^7^Department of Infectious Diseases, ASST Grande Ospedale Metropolitano Niguarda, Milan, Italy; ^8^Clinic of Infectious Diseases, Department of Health Sciences, ASST Santi Paolo e Carlo, University of Milan, Milan, Italy; ^9^Department of Emergency, ASST Spedali Civili University Hospital, Brescia, Italy; ^10^Department of Medical and Surgical Specialties, Radiological Sciences and Public Health, Università di Brescia, Brescia, Italy; ^11^Department of Electronics, Information and Bioengineering, Politecnico di Milano, Milan, Italy; ^12^Directorate General for Health, Milan, Italy; ^13^Fondazione IRCCS Ca' Granda Ospedale Maggiore Policlinico, Angelo Bianchi Bonomi Hemophilia and Thrombosis Center, Milan, Italy

**Keywords:** COVID-19, administrative databases, institutionalization, post-acute care, nursing home

## Abstract

**Background:**

To obtain the rate of admission to nursing homes (NHs) and to evaluate clinical characteristics and mortality rates of patients admitted to NHs after hospitalizations for COVID-19, compared to non-COVID-19 acutely hospitalized patients.

**Methods:**

We analyzed administrative data from Lombardy, a Northen Italian region, in individuals aged ≥50 years who were hospitalized and discharged alive in 2018 for acute conditions or, between February 2020 and June 2022, for COVID-19. Outcomes included NH institutionalization rates within 180 post-discharge day and mortality following NH admission. Kaplan–Meier curves and Cox proportional hazard models adjusted for age, sex, and comorbidities were used to assess the risks.

**Results:**

Among 133,216 COVID-19 hospitalizations in 2020–2022 and 239,099 acute hospitalizations in 2018, institutionalization rates within 180 post-discharge days were similar (3.7% for both cohorts). However, COVID-19 patients had higher adjusted risks of institutionalization (HR 1.70; 95% CI 1.63–1.78) and mortality within 6 months after NH admission (HR 2.08; 95% CI 1.90–2.27). Differences were more pronounced when considering patients hospitalized during the first COVID-19 pandemic wave.

**Conclusion:**

COVID-19 hospitalization significantly increases the risks of admission to NHs and early mortality after institutionalization in older individuals compared to hospitalizations due to other acute conditions.

## 1 Introduction

Functional and cognitive impairments, which often prevail among older individuals, are predictors of the degree of an individual’s frailty, which is in turn associated with increased vulnerability to adverse clinical, psychological and social outcomes (1). Ageing, frequently linked to multimorbidity, is also associated with higher risks of hospitalization, disability and need for daily assistance (2). Hospitalization itself, regardless of the acute condition, often represents a critical event by creating a vicious circle that in older adults results in permanent functional and cognitive decline (3). Hospital stays of frail and older individuals are frequently complicated by such additional factors such as delirium, nosocomial infections, immobility and the psychological toll of isolation, all of which exacerbate pre-existing frailty (4). At the time of hospital discharge these patients are often in a significant worse condition than at the time of admission, many of them being even unable to regain their prior levels of independence. This decline results in the need for long-term care in nursing homes (NHs) or rehabilitation facilities or the need for full-time caregiving at home. This transition poses substantial challenges to public health and social care systems (5).

Hospitalization of COVID-19 was no exception to the forementioned issues. Older adults, particularly those with pre-existing frailty and multimorbidity, have been the most vulnerable to severe outcomes from SARS-CoV-2 infection, including prolonged hospitalizations, need for intensive care and mortality (6). The immediate effects of COVID-19 on hospitalization rates and clinical outcomes in these individuals are well known but much less are known the long-term consequences after the acute phase of illness. Recent research focused extensively on the phenomenon of the post-COVID-19 condition, colloquially known as “long COVID,” which includes a spectrum of prolonged symptoms and clinical conditions following the initial infection (7), potential preventive strategies as vaccines and therapeutics interventions may help to mitigate these long-term effects (8). However, the specific impact of COVID-19 hospitalizations on the post-acute recovery of older individuals remains poorly explored.

The decision to institutionalize older patients after an acute hospital stay places a considerable burden on both healthcare systems and families. This issue has been particularly pronounced during the COVID-19 pandemic, when an apparently higher post-hospitalization institutionalization rate was observed among patients with severe COVID-19, particularly in those who developed pneumonia or respiratory failure (9). With this in mind, we chose to answer the important public health question on whether or not COVID-19 itself leads to a higher risk of institutionalization in older adult patients, whether this burden is comparable to that of other acute conditions requiring hospitalization and whether COVID-19 increases this risk compared to other acute conditions (10).

With this background and gaps of knowledge, the primary objective of this study was to assess NH admission rates among patients hospitalized for COVID-19 compared with those acutely hospitalized for other reasons in 2018, i.e., the year prior to the COVID-19 pandemic. The secondary objectives were (1) to study the clinical characteristics and mortality rates of patients admitted to NHs following hospitalization for COVID-19 with those admitted for non-COVID-19 causes, and (2) to assess the impact of hospitalization for COVID-19 compared to other causes on subsequent NH admissions. To explore these matters, we leveraged administrative databases from Lombardy, Northern Italy.

## 2 Methods

### 2.1 Study setting

Data were obtained from the administrative database of Lombardy, a Northern Italian region with a population of approximately 10 million inhabitants, of whom 23% are 65 years or older (11), one of the highest rates in the European Union (12). Notably, it was the first region in Europe to be heavily affected by the COVID-19 pandemic and experienced the highest rate of mortality during the early months of the outbreak with more than 10,000 recorded deaths (13).

### 2.2 Study cohorts

The study included two cohorts: (1) patients aged 50 years and older acutely hospitalized in 2018 for an array of reasons before the onset of the pandemic (group 1) and (2) patients aged 50 years and older hospitalized for COVID-19 from February 2020 until June 2022 (group 2). We excluded patients under 50 years of age since their probability to be institutionalized is very low. For group 1, only patients admitted through the emergency network or referred for hospitalization by general practitioners were included, with the exclusion of cases whose hospitalizations were scheduled or occurred in day-hospitals. Subjects who died in hospital, were already residents in NHs prior to hospital admission, or were institutionalized 180 days after hospital discharge were also excluded.

### 2.3 Data source

In Lombardy, healthcare is mainly public funded and data related to hospitalizations, drug prescriptions, and medical procedures are collected for administrative and reimbursement purposes. The Lombardy administrative database that collects healthcare data for all residents is being regularly updated by the Welfare Directorate to obtain and record information for administrative, claims and public health purposes on the use of health-care facilities by all region residents, irrespective of age, gender, ethnicity, income, and demographic characteristics. The generated database contains demographic information (biological sex, age), hospital data (admission and discharge date, main comorbidities and interventions), and drug dispensations (coded using the Anatomical Therapeutic Chemical classification system: ATC) for individuals not residing in NHs. Hospital diagnoses are coded using ICD-9-CM by patient’s ward physicians and successively validated by employees specifically trained in disease coding. The Drug Derived Complexity Index (DDCI) (14), based on drug prescriptions 1 year before hospitalization, was used as a proxy to assess comorbidities. After NHs admission, data were obtained and became available from Lombardy’s SOSIA [PMM: write at least once SOSIA in full] classification system (15). This database includes clinical and functional measures such as the Cumulative Illness Rating Scale (CIRS) index (16), selected Basic Activities of Daily Living (BADL), items from the Barthel index (17) and those relative to cognitive and behavioural conditions from the Gottfries, Brane and Steen scale (GBS) (18). Additional data included the presence of bedsore injury, catheter use, falls and physical restraints. From the CIRS two indices were calculated: a Severity Index (SI), determined as a mean from the first 13 items, and a Comorbidity Index (CI), calculated as a count of moderate or severe comorbidities. Furthermore, a short version of the GBS (sGBS) scale was derived from the available data, summarizing key behavioural aspects such as confusion, irritability and restlessness. Similarly, a short version of the BADL (sBADL) index summarized the items of chair-bed transfers, feeding capacity, personal hygiene, ambulation or wheelchair use. A mobility and behaviour index were also calculated according to the indicators in SOSIA. Mortality for individuals hospitalized in 2018 was censored at February 28, 2020 when the COVID-19 pandemic began. All data for analyses are secondary data primarily used for administrative purposes. Data are made available for analysis thanks to an agreement between the Lombardy Region and Mario Negri Institute (19) with the goal to evaluate appropriateness in drug use and analysis of treatment paths.

### 2.4 Ethics

All data were handled according to the current Italian law on privacy. In Italy, studies using aggregated, anonymous data from administrative databases that do not involve direct access to identifying information do not require Ethics Committee/IRB approval or notification, nor is a patient informed consent warranted.

### 2.5 Statistical analysis

Population characteristics were presented as numbers with percentages for categorical variables and means with standard deviations for continuous ones. The differences between the two cohorts were tested with a chi-square test for categorical variables and a t-test for continuous ones. Patients discharged alive from the hospital were followed for 180 days to check for any admission to a NH, starting on day 1. This admission and the mortality risk after entering NHs were shown by mean of Kaplan–Meier curves, and log-rank tests were performed. Cox Proportional Hazard models were fitted and Hazard Ratios (HRs) with 95% confidence interval (95%CIs) were reported. Models for NH admission were adjusted for age, biological sex and DDCI, while models for mortality were adjusted for age, sex, and comorbidity index. To further investigate the specific impact of COVID-19 on NH admissions, an additional Cox Proportional Hazard model, with adjustments for the same covariates, was employed comparing COVID-19 hospitalizations (overall and across different pandemic waves) to hospitalizations for diagnoses other than COVID-19. We also calculated the incidence rate of mortality with a 95%CI. Since the first wave of COVID-19 (from 20 February 2020 to 31 May 2020) was characterised by a strict lockdown and a stop to NH admissions and visits of relatives from March to June, we performed a sensitivity analysis excluding this period. Statistical analyses were performed using SAS software, SAS Institute Inc., Cary, NC, USA.

## 3 Results

### 3.1 Patient characteristics and NH admissions

**Figure 1:**
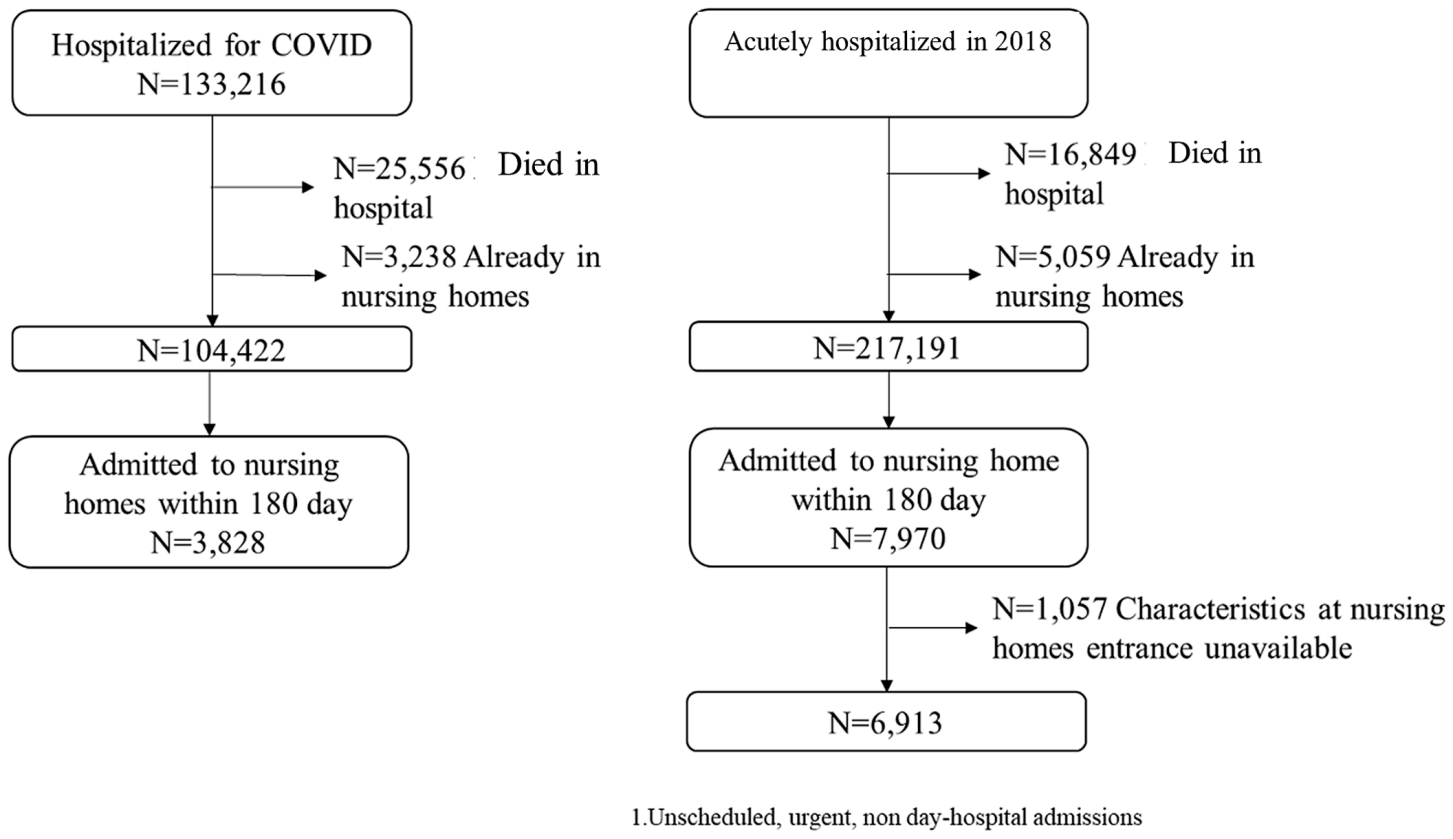
Flow-chart of the study.

Between February 20 2020 and June 30 2022, a total of 133,216 persons aged 50 or older were hospitalized due to COVID-19 (Figure 1). Among them, approximately one in five died during hospitalization and 2.4% were excluded from further analysis because already institutionalized. For comparison, in 2018 239,099 people were hospitalized for an array of unscheduled acute reasons. Of these, 7% died in hospital and 2.1% were excluded owing to their residence in NHs prior to hospital admission. The two final sample are 104,422 and 217,191, respectively for COVID-19 patients and the 2018 cohort. Therefore, the cohort analysed for incident NH admissions within 180 days of discharge included 3,828 COVID-19 hospitalized patients (3.7% of those alive at discharge and not already residing in NHs) and 7,970 cases hospitalized in 2018 (3.7% of those alive at discharge and not residing in NHs). Due to incomplete data, 1,057 cases were excluded (Figure 1). Patients hospitalized for COVID-19 were younger and with a higher proportion of men than the 2018 cohort (Table 1A). In the 2018 cohort, a higher proportion of cases (13.6% vs. 8.5%) died within 6 months without being admitted to NHs in association with higher DDCI comorbidity scores (*p*-values < 0.0001 for both comparisons).

**Table 1:** Characteristics of patients hospitalized due to COVID-19 and other causes.

	A. COVID-19 vs. 2018 hospitalizations			B. COVID-19 first wave vs. following waves		
	COVID-19	Hospitalized in 2018	*p*-value	First wave	Following waves	*p*-value
*N*	104,442	217,191		27,187	77,235	
Biological sex, *N* (%)			<0.0001			<0.0001
M	59,922 (57.4%)	110,880 (51.1%)		16,245 (59.8%)	43,677 (56.6%)	
F	44,500 (42.6%)	106,311 (48.9%)		10,942 (40.2%)	33,558 (43.4%)	
Age, mean (SD)	70.5 (11.8)	75.0 (11.9)	<0.0001	68.8 (11.4)	71.1 (11.9)	<0.0001
<75, *N* (%)	63,332 (60.7%)	94,242 (43.4%)		18,178 (66.9%)	45,154 (58.5%)	
75–84, *N* (%)	27,098 (26.0%)	70,742 (32.6%)		6,354 (23.4%)	20,744 (26.9%)	
85+, *N* (%)	13,992 (13.4%)	52,207 (24.0%)		2,655 (9.8%)	11,337 (14.7%)	
Admitted to intensive care, *N* (%)	6,888 (6.6%)	9,447 (4.4%)	<0.0001	4,755 (6.2%)	2,133 (7.9%)	<0.0001
DDCI, mean (SD)	3.6 (3.4)	4.7 (3.6)	<0.0001	3.3 (3.3)	3.7 (3.4)	<0.0001
Days of hospitalization, mean (SD)	14.7 (17.4)	10.9 (9.7)	<0.0001	15.8 (18.0)	14.4 (17.2)	<0.0001
Previous years hospitalizations, *N* (%)	24,640 (23.6%)	58,321 (26.9%)	<0.0001	6,298 (23.2%)	18,342 (23.7%)	0.0516
Number of previous year hospitalization, mean (SD)	2.1 (5.8)	1.5 (4.2)	<0.0001	2.2 (6.1)	2.1 (5.7)	0.4214
Deceased within 180 days after discharge, not entering a NH, *N* (%)	8,904 (8.5%)	29,592 (13.6%)	<0.0001	2,163 (8.0%)	6,741 (8.7%)	<0.0001
Entering a NH within 180 days, *N* (%)	3,828 (3.7%)	7,970 (3.7%)	0.9585	669 (2.5%)	3,159 (4.1%)	<0.0001
Discharged from NH	759 (19.8%)	1,759 (22.1%)	0.0054	51 (7.6%)	708 (22.4%)	<0.0001
Days between hospital discharge and NH admission (from day 0)*, median (IQR)	41 (10;84)	45 (7;97)	<0.0001	82 (50;117)	32 (5;71)	<0.0001
Days between hospital discharge and NH admission (from day 1), median (IQR)	56 (28;95)	64 (32;113)	<0.0001	87 (58;120)	47 (24;86)	<0.0001

*Including subjects discharged directly to the NH; DDCI: Drug Derived Complexity Index; NH: Nursing home; IQR: inter-quartile range; SD: standard deviation.

**Figure 2:**
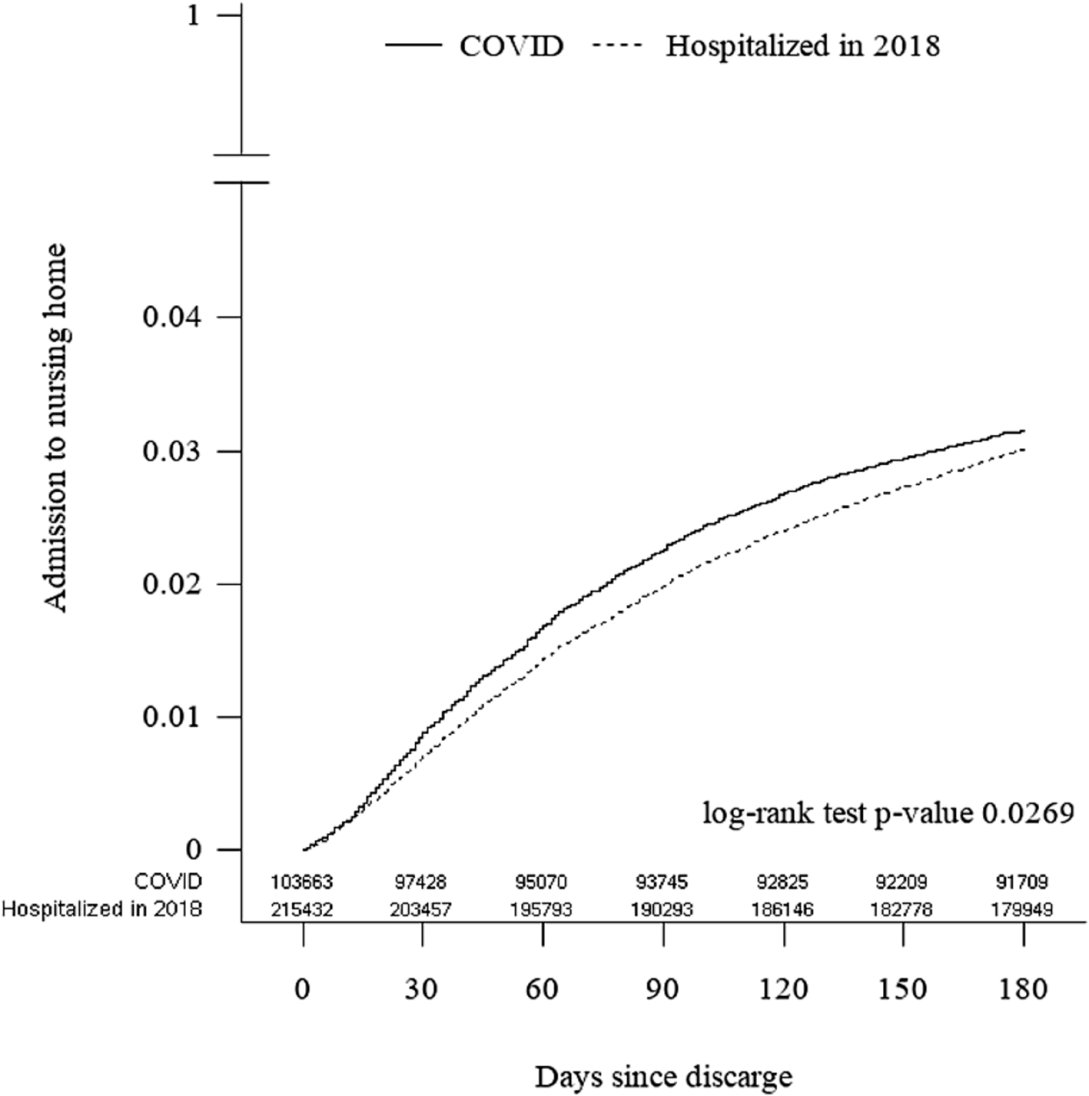
Nursing home admissions post-hospital discharge among patients hospitalized in 2018 and during COVID-19 pandemic.

Both cohorts were followed for 6 months and showed differences in NH admission rates (Figure 2). The univariate hazard ratio (HR) for NH admission in the COVID-19 cohort was 1.05 (95% CI 1.01–1.10) compared to the 2018 cohort. This HR increased to 1.70 (95% CI 1.63–1.78) after adjusting for age, sex, and DDCI scores.

Supplementary Table S1 lists the diagnoses leading to hospitalization with or without subsequent NH admission for the 2018 cohort. Supplementary Table S2 shows the impact of these diagnoses on NH admission rates compared to COVID-19 hospitalizations. Dementia was among the strongest predictors of NH institutionalization. Specifically, dementia showed an adjusted HR of 2.04 (95% CI 1.90–2.21) compared to COVID-19 hospitalizations, with a slightly higher risk during the first viral wave (2.15, 95% CI 2.01–2.31) than subsequent waves (1.95, 95% CI 1.80–2.10).

At NH admission, baseline evaluations revealed similar characteristics between the two cohorts, except for a higher prevalence of bedsores and urinary catheterization in the COVID-19 cohort (Table 2A). In this, there were more men (38% vs. 31%) and cases were on average 1 year younger. Approximately half of the institutionalized cases were aged 85 or older. Symptoms associated with dementia were prevalent in both groups, with delirium present in almost nine of ten cases. Restlessness was more frequent in the COVID-19 cohort. There were no significant differences between the two cohorts for comorbidity, mobility or behaviour index. However, more cases from the 2018 cohort required a wheelchair or a cane (*p* < 0.0001). Table 3 shows the HR for the risk of NH admission within 6 months post-hospital discharge in the different groups of patients.

**Table 2:** Characteristics of individuals at nursing home admission.

	A. COVID-19 vs. Other 2018 hospitalizations			B. COVID-19 first wave vs. COVID-19 subsequent waves		
	COVID-19	Hospitalized in 2018	*p*-value	First wave	Following waves	*p*-value
*N*	3,828	6,913		669	3,159	
Biological sex			<0.0001			0.5280
Males	1,443 (37.7%)	2,132 (30.8%)		245 (36.6%)	1,198 (37.9%)	
Females	2,385 (62.3%)	4,781 (69.2%)		424 (63.4%)	1961 (62.1%)	
Age, mean (sd)	83.6 (8.2)	84.7 (7.6)	<0.0001	82.9 (8.2)	83.8 (8.2)	0.0056
50–74	479 (12.5%)	641 (9.3%)		88 (13.2%)	391 (12.4%)	
75–84	1,372 (35.8%)	2,293 (33.2%)		259 (38.7%)	1,113 (35.2%)	
85+	1,977 (51.6%)	3,979 (57.6%)		322 (48.1%)	1,655 (52.4%)	
Severity index, mean (SD)	2.2 (0.4)	2.2 (0.4)	0.1675	2.1 (0.4)	2.2 (0.4)	0.0194
Comorbidity index, mean (SD)	6.0 (2.3)	6.1 (2.2)	0.0764	5.9 (2.3)	6.0 (2.3)	0.0631
sBADL, mean (SD)	12.6 (11.9)	13.1 (11.6)	0.0421	13.8 (12.4)	12.4 (11.8)	0.0042
sGBS, mean (SD)	5.8 (4.0)	5.6 (4.0)	0.0460	5.8 (3.9)	5.8 (4.0)	0.9231
Confusion	3,364 (87.9%)	6,068 (87.8%)	0.8768	582 (87.0%)	2,782 (88.1%)	0.4410
Irritability	1,577 (41.2%)	2,843 (41.1%)	0.9429	288 (43.0%)	1,289 (40.8%)	0.2838
Restlessness	1,569 (41.0%)	2,617 (37.9%)	0.0014	282 (42.2%)	1,287 (40.7%)	0.5000
Bedsores injury	786 (20.5%)	1,130 (16.3%)	<0.0001	121 (18.1%)	665 (21.1%)	0.0847
Urinary catheter	1,033 (27.0%)	1,370 (19.8%)	<0.0001	136 (20.3%)	897 (28.4%)	<0.0001
Falls	204 (5.3%)	413 (6.0%)	0.1688	41 (6.1%)	163 (5.2%)	0.3109
Physical restraints (mainly bed rails)	1,530 (40.0%)	2,839 (41.1%)	0.2668	251 (37.5%)	1,279 (40.5%)	0.1544
Walking aids			<0.0001			0.0108
No	621 (16.2%)	829 (12.0%)		124 (18.5%)	497 (15.7%)	
Cane/bow	563 (14.7%)	1,100 (15.9%)		118 (17.6%)	445 (14.1%)	
Artificial limb	3 (0.1%)	5 (0.1%)		-	3 (0.1%)	
Wheelchair	2,641 (69.0%)	4,979 (72.0%)		427 (63.8%)	2,214 (70.1%)	

(A) Comparison between all COVID patients and patients acutely hospitalized during 2018. (B) Comparison between patients hospitalized during COVID-19 first wave and following waves. sBADL: short Basic Activities of Daily Living (chair-bed transfers, feeding, personal hygiene, ambulation or wheelchair use); sGBS: short Gottfries, Brane and Steen scale (confusion, irritability, and restlessness).

**Table 3:** Nursing home admissions within 6 months post-hospital discharge in the two groups of patients.

	COVID vs Hospitalized HR (95% C.I.)	COVID-19 first wave vs. following waves HR (95% C.I.)
*N*	103,663; 215,432	27,136; 76,527
Unadjusted	1.05 (1.01; 1.10)	0.70 (0.64;0.77)
Age and sex adjusted	1.66 (1.59; 1.74)	0.93 (0.85;1.01)
Age, sex and DDCI adjusted	1.70 (1.63; 1.78)	0.92 (0.84;1.00)

Mortality within 18 months after NH admission was higher in the COVID-19 cohort, even after adjusting for sex, age, and CIRS comorbidity index (Table 4A). In the COVID-19 cohort, 1,670 patients died (incidence rate: 1.10‰, 95% CI 1.05–1.16), compared to 3,175 in the 2018 cohort (incidence rate: 1.00‰, 95% CI 0.96–1.03). This difference was driven by a higher mortality in the first 6 months after NH admission. There were 1,052 deaths in the COVID-19 cohort (mortality incidence rate: 1.86‰, 95% CI 1.75–1.98) compared to 1,764 deaths in the 2018 cohort (mortality incidence rate: 1.45‰, 95% CI 1.38–1.52), with an adjusted HR of 2.08 (95% CI 1.90–2.27). After 6 months, there was no difference in mortality (adjusted HR: 0.99, 95% CI 0.90–1.10).

**Table 4:** Mortality risk of subjects admitted to the nursing homes presented as total and divided by the first semester of stay and the following months.

	COVID-19 vs. 2018 hospitalization HR (95% C.I.)			COVID-19 first wave vs. following waves HR (95% C.I.)		
	All	0–180	181+	All	0–180	181+
Unadjusted	1.11 (1.05; 1.18)	1.28 (1.18;1.38)	0.91 (0.83;1.00)	0.70 (0.61;0.80)	0.69 (0.58;0.82)	0.71 (0.58;0.86)
Age and sex adjusted	1.12 (1.05; 1.19)	1.28 (1.19;1.38)	0.91 (0.83;1.01)	0.71 (0.62;0.81)	0.71 (0.59;0.85)	0.71 (0.58;0.87)
Age, sex, and comorbidity index adjusted	1.48 (1.39; 1.58)	2.08 (1.90; 2.27)	0.99 (0.90; 1.10)	0.72 (0.63;0.82)	0.72 (0.60;0.86)	0.71 (0.58;0.87)

(A) Comparison between all COVID patients and patients acutely hospitalized during 2018. (B) Comparison between patients hospitalized during COVID-19 first wave and following waves. The Hazard ratios compare the mortality of COVID-19 group with the 2018 hospitalization group, and the first COVID-19 wave to subsequent waves.

### 3.2 COVID-19 waves differential analysis

The first COVID-19 wave (hospital admission between February 20, 2020, and May 31, 2020) was more severe than subsequent waves, with NH admissions halted in late March 2020 and gradually resumed thereafter. During this wave, male patients were prevalent, and their average age was slightly lower compared to that of cases in subsequent waves (Table 1B). The NH admission rate during the first wave was also lower (2.5% vs. 4.1%). At NH admission, patients from the first wave were largely comparable to those from subsequent waves, even though they were slightly younger (less than a one-year difference) and had a lower prevalence of urinary catheterization (20.3% vs. 28.4%; Table 2B). Dementia symptoms were similarly prevalent across all waves, delirium being very common. The adjusted HR for mortality indicated a lower probability of death for patients hospitalized during the first wave (0.79, 95% CI 0.63–0.82; Table 3B).

## 4 Discussion

In the present analysis we found that patients hospitalized for COVID-19 had a higher risk of NH admission within 6 months after discharge, even after adjusting for age, biological sex and comorbidities. These findings underscore the substantial burden of COVID-19 on older people and highlight a critical issue in long-term care planning. Although older age and multimorbidity are generally associated with poor outcomes following hospitalization for acute illnesses (20), COVID-19 appears to uniquely exacerbate this risk, thus raising important public health concerns. This finding is likely due to the inflammatory and thrombotic effects of SARS-CoV-2 infection (21), the prolonged length of hospital stay associated with COVID-19 (22), as well as such hospitalization-associated complications as severe respiratory failure and prolonged immobility (23). Furthermore, the prolonged inflammatory response and catabolic state induced by severe SARS-CoV-2 infections is likely to have contributed to significant muscle mass loss and cachexia, leading to long-term functional impairment and increased morbidity (24). This impact on lean body mass may persist after hospital discharge, further complicating recovery and rehabilitation (25). Thus, it is not surprising that COVID-19 patients are more likely to present bedsore injuries and need of urinary catheterization (26, 27). In addition, restlessness was more prevalent in the COVID-19 group, suggesting a potential link between COVID-19 and post-infection neurocognitive symptoms contributing to restlessness (28, 29).

In addition to these clinical and functional consequences, public health policies implemented during the pandemic—particularly the no-visitation rules enforced in Italian nursing homes—may have had unintended negative consequences on resident mental health. Although these measures aimed to contain the spread of SARS-CoV-2, they also led to prolonged social isolation, reduced caregiver involvement, and worsened mental health among older adults. Several studies have highlighted that these restrictive practices were associated with accelerated cognitive and functional decline, depression, and increased behavioral disturbances (28). These indirect effects may have contributed to the excess early mortality and the need for institutionalization observed in the COVID-19 cohort, emphasizing the complex interplay between infection severity and environmental factors in shaping long-term outcomes.

However, higher risk of NH admission may also be related to changes in threshold for NH admission during pandemic due to less investment or willingness to invest from family members or other changes in health policy.

While most of the research and public health efforts during the pandemic focused on mitigating the acute effects of SARS-CoV-2 infection, particularly for preventing severe illness and mortality among frail individuals (30), less attention has been given to long-term consequences. Additionally, while it is well documented that the post-COVID-19 condition is a significant issue (31), the broader but more subtle consequences extending beyond hospital discharge, such as loss of autonomy and increased rate of institutionalization, received limited attention. Addressing these long-term outcomes is crucial from a public health perspective, because they influence the demand for long-term care services and the allocation of healthcare resources. The development of policies aimed at preserving independence and improving the quality of life in older adults will be crucial to identify strategies designed to improve recovery by integrating physical, pulmonary and cognitive rehabilitation into the post-discharge care plans (32, 33), considering that minimizing the need for long-term care will be undoubtedly beneficial for patient health and reduce the burden on the healthcare system.

We also found that dementia was associated with a higher risk of NH admissions compared to COVID-19 across all pandemic waves, particularly during the first one. This finding suggests that dementia, already known for its association with functional decline and loss of autonomy (34), creates a substantial burden on the health care system in terms of long-term care needs, even higher than COVID-19 itself. Models that include enhanced rehabilitation services may be especially relevant during health crises like a pandemic, when vulnerable populations with multiple chronic conditions are disproportionately affected. Indeed, sudden health emergencies can significantly increase the institutionalization risk, which is already high for the acute illness itself even in the absence of pre-existing conditions.

In our cohort, COVID-19 patients experienced a significantly higher mortality rate within the first 6 months following NH admission, with an adjusted HR more than double that of the 2018 cohort taken for comparison. The early phases of the pandemic were characterized by exceptionally high mortality rates, particularly among older individuals (35), largely due to limited therapeutic options, absence of vaccines and overwhelmed healthcare systems that had to face critical resource shortage of resources, including ICU beds (36). Nonetheless, our finding of a gradual convergence of long-term mortality rates between the two investigated cohorts suggests a dynamic interplay of factors that helped to mitigate the initially devastating outcomes. Expanded vaccine coverage and natural immunity through infection to have redefined the trajectory of COVID-19-related mortality (37).

The comparison between the first and subsequent pandemic waves further enriched our analysis. The first wave was characterized by extreme lethality and reduced NH admissions that reflected both the novelty and severity of the virus in an unprepared region setting. In contrast, later waves demonstrated a shift in the balance, with increased NH admissions and lower mortality rates, indicative of a more resilient health care response, a population partially shielded by increased immunity and refined medical interventions. We believe that this perspective not only emphasizes the importance of preparedness and adaptability in managing global health emergencies but also raises critical questions about resilience in healthcare systems and equity in access to life-saving resources.

Furthermore, triage strategies implemented during pandemic surges likely played a role in shaping patient trajectories. In the overwhelmed healthcare systems such as that of Lombardy during the first wave, clinicians were often forced to prioritize patients based on their likelihood of recovery and potential for positive functional outcomes. Frail older adults with severe COVID-19 and high expected post-recovery disability were less frequently admitted to intensive care units, in alignment with ethical frameworks aimed at avoiding therapeutic obstinacy. This selective ICU admission pattern may have influenced not only survival but also post-hospital functional status and institutionalization rates, as many patients received less aggressive interventions and fewer opportunities for intensive rehabilitation.

Specifically in Italy NHs operate under a mixed public-private model (9) and families are typically responsible for room and board expenses. This system may lead to disparities in access to long-term care, particularly for socioeconomically disadvantaged people. Therefore, public health strategies should focus on expanding affordable rehabilitation programs, strengthening home care services and providing financial assistance to reduce the burden on families and ensure more equitable access to essential care services. Moreover, national surveillance programs might help to monitor post-COVID functional decline and support early interventions for those at risk of institutionalization (38). Integrating these insights into broader public health frameworks would help to improve the health care system resilience and foster equity in access to care.

The strengths of this study lie in the robust use of a large administrative database from Lombardy, allowing for a comprehensive analysis of real-world big data. However, administrative data may introduce inaccuracies related to coding. The COVID-19 pandemic itself, which disrupted the health care systems and altered admission practices, may have influenced the observed differences in outcomes, making comparisons with the pre-pandemic 2018 cohort challenging. The management of the pandemic substantially changed across the years analyzed in our study, giving us an uncertain picture of COVID-19 outcomes: after a period in which the greatest part of hospitals was converted to care only for COVID-19 patients and NHs did not accept new subjects and even shut down visits by relatives, the situation returned to quasi-normality on the following waves. However, to partially counterbalance this instability, we also included two separated analyses for selected outcomes. Third, our study was based in Lombardy that, while representative of high-income European regions, may not reflect outcomes in settings with different demographics, healthcare infrastructures, and pandemic responses.

## 5 Conclusion

The present study shows that COVID-19 hospitalization adversely impacted long-term outcomes in frail older adults, leading to increased rates of institutionalization and early mortality. These effects extended beyond the acute phase and put emphasis on the importance of considering not only the immediate clinical management but also the long-term care needs of older COVID-19 patients. Given the high burden on the health care systems, targeted interventions aimed at improving post-acute recovery, preserving autonomy and reducing the risk of institutionalization are urgently needed. Understanding these outcomes is crucial to better support vulnerable populations in the post-acute recovery phase and to strengthen home-based care services. These findings also support other preventive measures, such as vaccination and early implementation of other prevention policies. Furthermore, these results highlight the importance of researching into the broader implications of COVID-19 on aging populations, particularly in the context of evolving health care challenges and persistent risks posed by emerging infectious diseases. We also highlight the necessity of increasing regional and global resilience towards the long-term consequences of severe acute illnesses by improving health care facilities and policies, in order to reduce institutionalization risk and improve equitable access to long-term care.

## Data Availability

Data that support the findings of this study are available by the Lombardy Region. Data are not publicly available due to restrictions linked to privacy regulation. Requests to access the datasets should be directed to alessandro.nobili@marionegri.it.
