# Supplementary material for: The impact of COVID-19 hospitalizations on nursing home admissions: a regional insight into long-term care and public health
Source: Front Public Health. 2025 Jul 18;13:1613684. doi: 10.3389/fpubh.2025.1613684 (PMC12315697; doi:10.3389/fpubh.2025.1613684)
Supplement: Supplementary file 1 [file Table_1.docx]

**Supplementary materials**

**Table S1: First diagnosis of hospitalization by International Classification of Diseases 9th revision**

| **Admission into a nursing home within 6 months** | **No** | **Yes** |
| --- | --- | --- |
| **N** | 309,815 | 11,798 |
| **COVID** | 100,594 (32.5%) | 3,828 (32.5%) |
| **First wave** | 26,518 (11.3%) | 669 (7.7%) |
| **Other** | 74,076 (26.2%) | 3,159 (28.4%) |
| **Infectious and parasitic diseases (001-139)** | 7,367 (2.4%) | 310 (2.6%) |
| **Neoplasms** **(140-239)** | 13,761 (4.4%) | 195 (1.7%) |
| **Endocrine, nutritional and metabolic diseases, and immunity disorders** **(240-279)** | 3,556 (1.2%) | 206 (1.8%) |
| **Diseases of blood and blood-forming organs** **(280-289)** | 3,524 (1.1%) | 111 (0.9%) |
| **Mental disorders (290-319)** | 4,112 (1.3%) | 248 (2.1%) |
| **Diseases of the nervous system and sense organs** **(320-389)** | 5,888 (1.9%) | 191 (1.6%) |
| **Diseases of the circulatory system (390-459)** | 59,654 (19.3%) | 2,001 (17.0%) |
| **Diseases of the respiratory system (460-519)** | 29,230 (9.4%) | 1,278 (10.8%) |
| **Diseases of the digestive system (520-579)** | 26,007 (8.4%) | 457 (3.9%) |
| **Diseases of the genitourinary system (580-629)** | 12,898 (4.2%) | 515 (4.4%) |
| **Diseases of the skin and subcutaneous tissue** **(680-709)** | 1,231 (0.4%) | 57 (0.5%) |
| **Diseases of the musculoskeletal system and connective tissue** **(710-739)** | 2,668 (0.9%) | 123 (1.0%) |
| **Symptoms, signs, and ill-defined conditions** **(780-799)** | 8,222 (2.7%) | 183 (1.6%) |
| **Injury and poisoning** **(800-999)** | 29,608 (9.6%) | 2,069 (17.5%) |
| **Remaining** | 1,495 (0.5%) | 26 (0.2%) |
| - **Complications of pregnancy, childbirth, and the puerperium** **(630-677)** | 45 | - |
| - **Congenital anomalies** **(740-759)** | 182 | 1 |
| - **Supplementary classification of factors influencing health status and contact with health services (V codes)** | 1268 | 25 |
