# Supplementary material for: The impact of COVID-19 hospitalizations on nursing home admissions: a regional insight into long-term care and public health
Source: Front Public Health. 2025 Jul 18;13:1613684. doi: 10.3389/fpubh.2025.1613684 (PMC12315697; doi:10.3389/fpubh.2025.1613684)
Supplement: Supplementary file 2 [file Table_2.docx]

**Table S2. Risk of admission to the nursing homes within 6 months after hospitalization.**

**A: All subjects hospitalized for COVID, B: Subjects hospitalized for COVID during first wave (February-June 2020), C: Subjects hospitalized for COVID after first wave (July 2020-June 2022).**

| First diagnosis of hospitalization | Unadjusted | P-value | Age and sex adjusted | P-value | Age, sex and ddci adjusted | P-value |
| --- | --- | --- | --- | --- | --- | --- |
| A. COVID ALL | ref |  | ref |  | ref |  |
| Infectious and parasitic diseases | 1.01 (0.88;1.16) | 0.8657 | 0.65 (0.57;0.74) | <.0001 | 0.62 (0.54;0.71) | <.0001 |
| Neoplasms | 0.44 (0.37;0.52) | <.0001 | 0.36 (0.31;0.43) | <.0001 | 0.37 (0.31;0.43) | <.0001 |
| Endocrine, nutritional and metabolic diseases, and immunity disorders | 1.22 (1.02;1.44) | 0.0274 | 0.67 (0.56;0.80) | <.0001 | 0.63 (0.53;0.75) | <.0001 |
| Diseases of blood and blood-forming organs | 0.82 (0.67;1.01) | 0.0666 | 0.43 (0.35;0.54) | <.0001 | 0.42 (0.34;0.52) | <.0001 |
| Mental disorders | 1.32 (1.13;1.53) | 0.0003 | 1.92 (1.65;2.23) | <.0001 | 1.86 (1.59;2.16) | <.0001 |
| Diseases of the nervous system and sense organs | 0.81 (0.68;0.95) | 0.0099 | 0.81 (0.69;0.95) | 0.0111 | 0.80 (0.68;0.95) | 0.0091 |
| Diseases of the circulatory system | 0.84 (0.79;0.90) | <.0001 | 0.51 (0.48;0.54) | <.0001 | 0.50 (0.47;0.53) | <.0001 |
| Diseases of the respiratory system | 1.07 (1.00;1.16) | 0.0520 | 0.58 (0.54;0.62) | <.0001 | 0.55 (0.51;0.59) | <.0001 |
| Diseases of the digestive system | 0.44 (0.39;0.49) | <.0001 | 0.33 (0.29;0.36) | <.0001 | 0.32 (0.29;0.36) | <.0001 |
| Diseases of the genitourinary system | 0.94 (0.85;1.05) | 0.2694 | 0.65 (0.59;0.73) | <.0001 | 0.63 (0.57;0.70) | <.0001 |
| Diseases of the skin and subcutaneous tissue | 1.15 (0.86;1.55) | 0.3509 | 0.88 (0.65;1.18) | 0.3913 | 0.83 (0.62;1.12) | 0.2217 |
| Diseases of the musculoskeletal system and connective tissue | 1.16 (0.95;1.42) | 0.1499 | 0.94 (0.77;1.14) | 0.5125 | 0.90 (0.73;1.10) | 0.2873 |
| Symptoms, signs, and ill-defined conditions | 0.57 (0.48;0.68) | <.0001 | 0.43 (0.37;0.51) | <.0001 | 0.43 (0.36;0.51) | <.0001 |
| Injury and poisoning | 1.72 (1.62;1.82) | <.0001 | 0.91 (0.86;0.97) | 0.0042 | 0.92 (0.86;0.98) | 0.0057 |
| Remaining | 0.45 (0.29;0.69) | 0.0003 | 0.46 (0.30;0.72) | 0.0006 | 0.45 (0.29;0.70) | 0.0004 |
| B. COVID first wave | ref |  | ref |  | ref |  |
| Infectious and parasitic diseases | 1.33 (1.15;1.55) | 0.0002 | 0.73 (0.63;0.85) | <.0001 | 0.71 (0.61;0.82) | <.0001 |
| Neoplasms | 0.58 (0.49;0.70) | <.0001 | 0.41 (0.34;0.49) | <.0001 | 0.41 (0.34;0.49) | <.0001 |
| Endocrine, nutritional and metabolic diseases, and immunity disorders | 1.60 (1.33;1.93) | <.0001 | 0.76 (0.63;0.92) | 0.0042 | 0.73 (0.60;0.88) | 0.0008 |
| Diseases of blood and blood-forming organs | 1.08 (0.87;1.35) | 0.4743 | 0.49 (0.39;0.61) | <.0001 | 0.48 (0.38;0.60) | <.0001 |
| Mental disorders | 1.74 (1.47;2.05) | <.0001 | 2.09 (1.77;2.47) | <.0001 | 2.04 (1.72;2.41) | <.0001 |
| Diseases of the nervous system and sense organs | 1.06 (0.89;1.27) | 0.5158 | 0.89 (0.74;1.06) | 0.1940 | 0.89 (0.74;1.06) | 0.1822 |
| Diseases of the circulatory system | 1.11 (1.01;1.22) | 0.0276 | 0.57 (0.52;0.63) | <.0001 | 0.56 (0.51;0.62) | <.0001 |
| Diseases of the respiratory system | 1.42 (1.28;1.57) | <.0001 | 0.66 (0.59;0.73) | <.0001 | 0.63 (0.57;0.70) | <.0001 |
| Diseases of the digestive system | 0.58 (0.51;0.66) | <.0001 | 0.36 (0.32;0.42) | <.0001 | 0.36 (0.32;0.41) | <.0001 |
| Diseases of the genitourinary system | 1.24 (1.09;1.41) | 0.0009 | 0.74 (0.65;0.84) | <.0001 | 0.72 (0.63;0.82) | <.0001 |
| Diseases of the skin and subcutaneous tissue | 1.52 (1.12;2.06) | 0.0073 | 0.99 (0.73;1.34) | 0.9234 | 0.94 (0.69;1.28) | 0.7057 |
| Diseases of the musculoskeletal system and connective tissue | 1.53 (1.23;1.89) | <.0001 | 1.04 (0.84;1.28) | 0.7237 | 1.01 (0.81;1.24) | 0.9616 |
| Symptoms, signs, and ill-defined conditions | 0.76 (0.63;0.91) | 0.0027 | 0.48 (0.40;0.58) | <.0001 | 0.48 (0.40;0.57) | <.0001 |
| Injury and poisoning | 2.26 (2.06;2.48) | <.0001 | 1.04 (0.94;1.14) | 0.4396 | 1.04 (0.95;1.14) | 0.4132 |
| Remaining | 0.59 (0.38;0.92) | 0.0199 | 0.51 (0.33;0.79) | 0.0029 | 0.50 (0.32;0.78) | 0.0024 |
| C. COVID waves after first | ref. |  | ref |  | ref |  |
| Infectious and parasitic diseases | 0.93 (0.81;1.06) | 0.2940 | 0.64 (0.56;0.73) | <.0001 | 0.61 (0.53;0.70) | <.0001 |
| Neoplasms | 0.40 (0.34;0.47) | <.0001 | 0.35 (0.30;0.42) | <.0001 | 0.35 (0.30;0.42) | <.0001 |
| Endocrine, nutritional and metabolic diseases, and immunity disorders | 1.12 (0.94;1.33) | 0.2133 | 0.66 (0.55;0.79) | <.0001 | 0.62 (0.52;0.74) | <.0001 |
| Diseases of blood and blood-forming organs | 0.76 (0.61;0.93) | 0.0090 | 0.43 (0.35;0.53) | <.0001 | 0.42 (0.34;0.51) | <.0001 |
| Mental disorders | 1.21 (1.04;1.41) | 0.0128 | 1.86 (1.60;2.17) | <.0001 | 1.81 (1.55;2.10) | <.0001 |
| Diseases of the nervous system and sense organs | 0.74 (0.63;0.87) | 0.0004 | 0.79 (0.67;0.93) | 0.0047 | 0.78 (0.66;0.92) | 0.0037 |
| Diseases of the circulatory system | 0.78 (0.73;0.83) | <.0001 | 0.50 (0.47;0.53) | <.0001 | 0.49 (0.46;0.52) | <.0001 |
| Diseases of the respiratory system | 0.99 (0.92;1.06) | 0.7436 | 0.57 (0.53;0.62) | <.0001 | 0.54 (0.50;0.58) | <.0001 |
| Diseases of the digestive system | 0.40 (0.36;0.45) | <.0001 | 0.32 (0.29;0.36) | <.0001 | 0.32 (0.28;0.35) | <.0001 |
| Diseases of the genitourinary system | 0.87 (0.78;0.96) | 0.0090 | 0.64 (0.58;0.71) | <.0001 | 0.62 (0.56;0.69) | <.0001 |
| Diseases of the skin and subcutaneous tissue | 1.06 (0.79;1.43) | 0.7048 | 0.86 (0.64;1.16) | 0.3228 | 0.82 (0.61;1.10) | 0.1803 |
| Diseases of the musculoskeletal system and connective tissue | 1.07 (0.87;1.30) | 0.5349 | 0.92 (0.75;1.12) | 0.3895 | 0.88 (0.72;1.08) | 0.2098 |
| Symptoms, signs, and ill-defined conditions | 0.53 (0.44;0.62) | <.0001 | 0.43 (0.36;0.50) | <.0001 | 0.42 (0.35;0.50) | <.0001 |
| Injury and poisoning | 1.58 (1.48;1.68) | <.0001 | 0.90 (0.85;0.96) | 0.0016 | 0.90 (0.85;0.96) | 0.0018 |
| Remaining | 0.41 (0.26;0.64) | <.0001 | 0.45 (0.29;0.70) | 0.0004 | 0.44 (0.28;0.69) | 0.0003 |
